# Decreased Risk of Anxiety in Diabetic Patients Receiving Glucagon-like Peptide-1 Receptor Agonist: A Nationwide, Population-Based Cohort Study

**DOI:** 10.3389/fphar.2022.765446

**Published:** 2022-02-23

**Authors:** Wen-Hsuan Tsai, Fung-Chang Sung, Lu-Ting Chiu, Ying-Hsiu Shih, Ming-Chieh Tsai, Shu-I Wu

**Affiliations:** ^1^ Division of Endocrinology and Metabolism, Department of Internal Medicine, Mackay Memorial Hospital, Taipei, Taiwan; ^2^ Management Office for Health Data (DryLab), Clinical Trial Research Center (CTC), China Medical University Hospital, Taichung, Taiwan; ^3^ Department of Health Services Administration, College of Public Health, China Medical University, Taichung, Taiwan; ^4^ Department of Food Nutrition and Health Biotechnology, Asia University, Taichung, Taiwan; ^5^ Department of Medicine, MacKay Medical College, New Taipei City, Taiwan; ^6^ Department of Psychiatry, Mackay Memorial Hospital, Taipei, Taiwan

**Keywords:** depression, anxiety, glucagon-like peptide-1 receptor agonist, diabetes, neuroendocrine

## Abstract

**Background:** Previous findings on using Glucagon-like peptide-1 receptor agonist (GLP1-RA) as an antidepressant were conflicting, lacking large-scale studies. We used population-based data to investigate depression and anxiety risk in diabetic patients receiving the medication.

**Methods:** From claims records of the National Health Insurance Research Database (NHIRD) of Taiwan, we identified cohorts of 10,690 GLP1-RA users and 42,766 propensity score-matched patients without GLP1-RA use from patients with diabetes mellitus (DM) diagnosed in 2011–2017, matched by age, gender, index year, occupation, urbanization, comorbidities, and medications. Incidence, hazard ratios (HR) and 95% confidence interval (CI) of depression and/or anxiety were estimated by the end of 2017.

**Results:** The overall combined incidence of anxiety and/or depression was lower in GLP1-RA users than in non-users (6.80 *versus* 9.36 per 1,000 person-years), with an adjusted HR adjusted hazard ratio (aHR) of 0.8 (95% CI: 0.67–0.95) after controlling for covariates. The absolute incidence reduction was greater in anxiety (2.13 per 1,000 person-years) than in depression (0.41 per 1,000 person-years). The treatment effectiveness was significant for women. Patients taking GLP1-RA for longer than 180 days had the incidence of anxiety reduced to 2.93 per 1,000 person-years, with an aHR of 0.41 (95%CI: 0.27–0.61), compared to non-users. Dulaglutide could significantly decrease risks of both anxiety and depression.

**Conclusion:** Patients with DM receiving GLP1-RA therapy have a greater reduction of the risk of anxiety than that of depression. Our findings strengthen previous research that advocated possible anti-depressant or anxiolytic effects of GLP1-RA and may lead to improved treatment adherence among patients with DM.

## Introduction

Diabetes mellitus (DM) is a group of endocrinological and metabolic disorders affecting approximately 422 million patients worldwide (Saeedi et al., 2019). Patients with DM are at an elevated risk of developing acute and serious long-term complications (Fowler, 2008; Gregg et al., 2016; Harding et al., 2019). Considerable evidence has also associated DM with increased risk of incident or recurrent depression (Gavard et al., 1993; Lustman et al., 2000; Anderson et al., 2001; Snoek et al., 2015; Chima et al., 2017; Khaledi et al., 2019). An earlier meta-analysis found that the risk of depression was 2-fold higher for patients with DM than individuals without DM (Anderson et al., 2001). Another meta-analysis review reported that poor glycemic control is associated with the occurrence of depression in patients with both type 1 and type 2 DM (Lustman et al., 2000).

Metformin, thiazolinediones (TZDs), glucagon-like peptide-1 receptor agonists (GLP1-RA), and dipeptidyl peptidase 4 (DPP-4) inhibitors are medications commonly prescribed for glycemic and weight control (Pozzi et al., 2019). A meta-analysis of clinical trials found that the use of pioglitazone, a TZD, was associated with significant improvements in depressive symptoms. The effect was more marked in women. The treatment effectiveness of metformin for diabetic patients was not consistent among studies (Moulton et al., 2018). However, a recent study in Saudi Arabia found that metformin could lower the probability of major depressive symptoms by 70% among patients with polycystic ovary syndrome (PCOS), but did not influence the occurrence of anxiety (AlHussain et al., 2020).

GLP1-RAs can cross the blood brain barrier, exerting function at both peripheral and central systems (Pozzi et al., 2019). A meta-analysis advocated that GLP1-RAs could exert antidepressant or anxiolytic effects on reducing the depression rating score of −2.09 (95% CI −2.28 – −1.91, *p* < 0.001) for diabetic patients (Pozzi et al., 2019). A United Kingdom study evaluating changes of quality of life for patients with GLP1-RA demonstrated that the therapy significantly reduced the Hospital Anxiety and Depression Scale (HADS) scores, compared with insulin-treated patients. (Grant et al., 2011). A placebo-controlled single-blind study, investigating the effect of GLP1-RA on excessive daytime sleepiness with 16 male DM patients, demonstrated a significant reduction in depression scores after the treatment compared to the baseline scores. Nonetheless, the reduction was not significant compared to placebo (Idris et al., 2013). A 26-weeks randomized controlled trial in the Netherlands found that GLP1-RA treatment in 26 patients significantly improved the quality of life measured by the Problem Areas in Diabetes Scale (PAID) questionnaire, compared to 24 patients using standard treatment (de Wit et al., 2014). Further follow-up study revealed that the effectiveness was sustained to 52 weeks, but there was insignificant improvement in scores of Beck Depression Index (BDI) (de Wit et al., 2016). Another 6-month treatment with liraglutide for 19 women with polycystic ovary syndrome without DM revealed no significant association with reduced depression, compared to 17 controls matched by age (Kahal et al., 2019). A pooled analysis on 5,325 individuals with higher BMI without diabetes evaluating the effectiveness of trials using GLP1-RA for 32–160 weeks also found no significant differences in depression (2.1 *versus* 2.1 events/100 person-years) and anxiety (1.9 *versus* 1.7 events/100 person-years) (O'Neil et al., 2017).

Most previous studies on GLP1-RA were undertaken with small sample sizes and reported inconsistent findings on the treatment effectiveness related to risks of depression and anxiety. These studies ascertained conditions of depression or anxiety mainly based on screening instruments rather than clinical diagnoses. A large long-term cohort study with sufficient sample size focusing on DM patients is lacking. Therefore, we used the insurance claims data of Taiwan to conduct this study to evaluate risks of anxiety and/or depression between DM patients with and without GLP1-RA, with sufficient large population-based cohorts and follow-up time.

## Methods and Materials

### Data Source

The National Health Insurance (NHI) of Taiwan is a compulsory health insurance system, launched in 1995 for all residents. For this study, we used the National Health Insurance Research Database (NHIRD) issued by NHI containing claims data of all insured individuals. The claims data provided records of birth date, gender, places of residence, and enrollee category, as well as diagnoses, drug prescriptions, and treatments for emergency and outpatient visits, and hospitalizations from 2000 to 2017. All identifications of insured people in the NHIRD were replaced with surrogate numbers and analyzed anonymously, hence consents were waived. This study was approved by the Institutional Review Board of Mackay Memorial Hospital (20MMHIS431e).

### Study Cohorts

**FIGURE 1:**
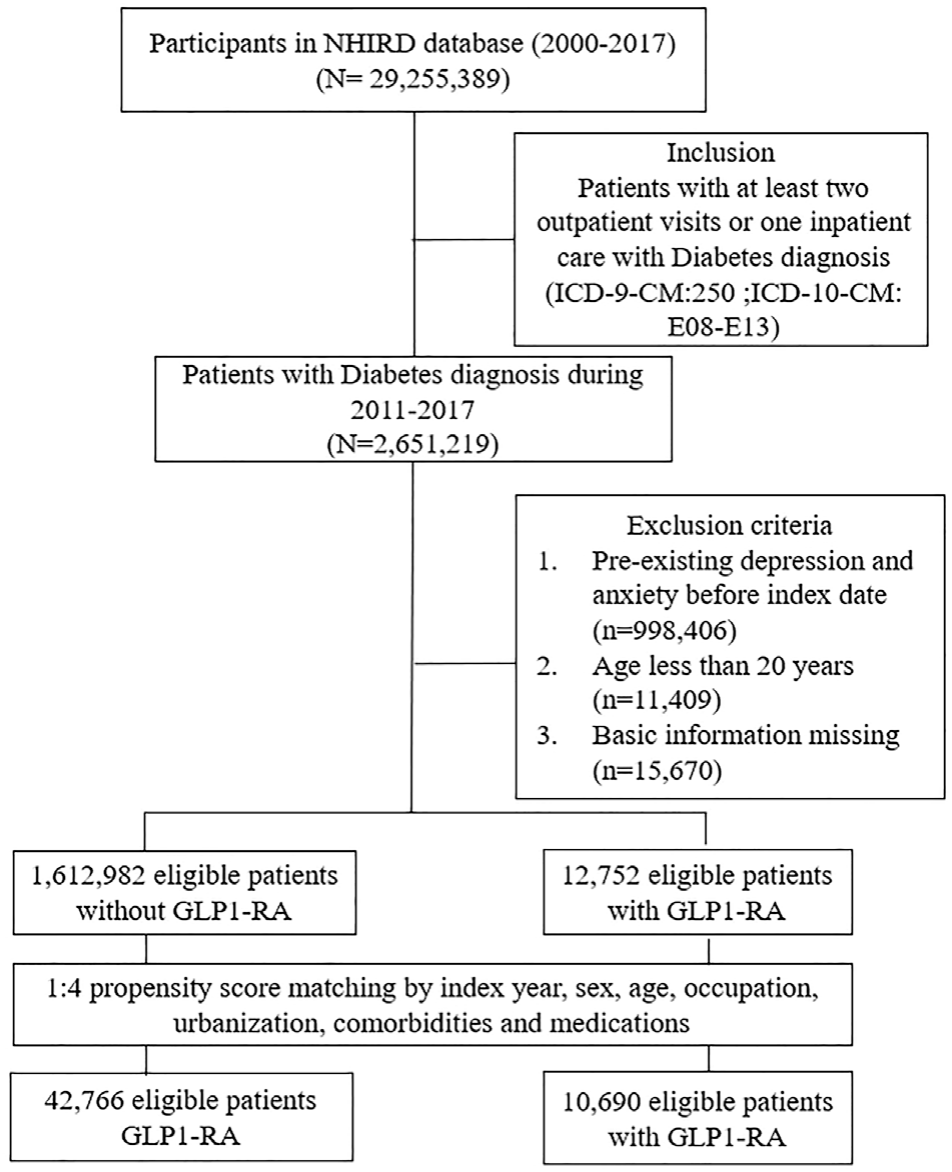
Selection scheme for study population.

GLP1-RA was one of the available treatment options for DM patients, with approval from the NHI given in 2011. We therefore first identified 4,079,299 patients with the diagnosis of DM during the period of 2011–2017 (Figure 1). Patients who were less than 20 years old or had been diagnosed with depression and/or anxiety at baseline were excluded. Patients who were newly diagnosed in 2011–2017 with DM (ICD-10-CM code E08, E09, E10, E11, and E13; ICD-9-CM codes: 250) at least twice were considered as the potential study population. The date of the initial diagnosis with DM was designated as the index date. Patients with DM prescribed with GLP1-RA (Liraglutide, Dulaglutide, Exenatide) in 2011–2017 were included in the GLP1-RA group. For the comparison group, propensity score-matched DM patients without GLP1-RA use were selected and matched at a 1:4 ratio. The propensity score was calculated by logistic regression using covariates of age, gender, index year, occupation, urbanization, comorbidities, and medications.

### Outcome

Both cohorts were followed to estimate follow-up person-years for each individual in both groups until incident depression (ICD-10-CM codes: F32.0, F32.1, F32.2, F32.3, F32.4, F32.5, F32.9, F33.0, F33.1, F33.2, F33.3, F33.40, F33.41, F33.42, F34.1, and F43.21; ICD-9-CM codes: 296.20, 296.21, 296.22, 296.23, 296.24, 296.25, 296.26, 296.30, 296.31, 296.32, 296.33, 296.34, 296.35, 296.36, 311, 300.4, 309.0, and 309.1) and/or anxiety (ICD-10-CM code: F41; ICD-9-CM codes: 300) were identified, or the individual withdrew from the insurance, or the end of 2017. Depression and anxiety were defined as having the diagnoses from at least two outpatient visits or one hospitalization to ensure the validity (Chien et al., 2007). The incidence rate of depression or anxiety was estimated by per 1,000 person-years.

### Potential Risk Covariates

Variables that might have an association with the development of depression or anxiety included comorbidities, other medications and demographic variables of occupation and urbanization level of residential area. We classified residential areas into four levels, with level 1 as the most urbanized, and level 4 as the least urbanized. We were interested in baseline comorbidities (Read et al., 2017), including hyperlipidemia (ICD-10-CM code: E78; ICD-9-CM codes: 272), hypertension (ICD-10-CM codes: I10, I11, I12, I13, and I15; ICD-9-CM codes: 401, 402, 403, 404, and 405), coronary artery disease (ICD-10-CM codes: I20, I21, I22, I23, I24, and I25; ICD-9-CM codes: 410, 411, 412, 413, 414, and 429), chronic kidney disease (ICD-10-CM codes: N18 and N19; ICD-9-CM codes: 585, 586, and 593), stroke (ICD-10-CM codes: I60, I61, I62, and I63; ICD-9-CM codes: 430, 431, 432, and 433), heart failure (ICD-10-CM code I50; ICD-9-CM codes: 428), chronic obstructive pulmonary disease (COPD) (ICD-10-CM codes: J44, J45, and J47; ICD-9-CM codes: 491, 493, 494, and 496), connective tissue disease (ICD-10-CM codes L93, L94; ICD-9-CM codes: 695 and 701), obesity (ICD-10-CM code E66; ICD-9-CM codes: 278), malignancy (ICD-10-CM codes D49; ICD-9-CM codes: 239), liver cirrhosis (ICD-10-CM code: K74; ICD-9-CM codes: 571), alcoholic liver disease (ICD-10-CM code: K70; ICD-9-CM codes: 571) and adapted Diabetes Complications Severity Index (aDCSI). The aDCSI is a good measure of diabetes severity that uniquely incorporates a wide range of diabetes complications (Young et al., 2008; Chang et al., 2012). The other anti-glycemic agents, including metformin, thiazolinedione (TZD), sodium-glucose co-transporters 2 (SGLT2) inhibitors, acarbose, sulfonylurea, dipeptidyl peptidase 4 (DPP-4) inhibitors, insulin, and compound agents, were also possible confounders to consider in the analyses.

### Statistical Analysis

Demographic characteristics and prevalence of comorbidities in the study and the comparison groups were compared and examined with Chi-square tests for categorical variables and *t*-tests for continuous variables. The Kaplan-Meier method was used to graphically describe the cumulative incidence of depression and anxiety during the 7-years follow-up period. Cox proportional hazards regression analysis was used to determine the GLP1-RA group to compare group hazard ratio (HR) and 95% confidence interval (CI) of depression or anxiety. Demographic variables, comorbidities, and medications were included in multivariable models to estimate the adjusted hazard ratio (aHR): Model 1 adjusted for demographic factors, Model 2 adjusted for Model 1 variables and comorbidities, and Model 3 adjusted for Model 2 variables and medications. We further evaluated the treatment effectiveness by the length of treatment, stratifying the medication courses into 3 periods, 30–90 days, 91–180 days, and >180 days. All statistical analyses were performed using STATA version 14.0 (StataCorp) and results with *p* values less than 0.05 considered as significant.

## Results

A total of 10,690 DM patients prescribed GLP1-RA and 42,766 comparisons with non-users were identified from the NHIRD. Both groups had similar distributions of age, gender, occupation, urbanization and comorbidities, with a mean age of 53.33 years (SD 13.04) and 45.06% women (Table 1). Although, the percentage of using metformin was higher in non-GLP1-RA users.

**TABLE 1:** Baseline demographic factors and comorbidities of study population according to GLP1-RA use.

Characteristics	Non-GLP1-RA user N = 42766		GLP1-RA user N = 10690		*p*-value	SMD
	n	%	n	%		
Gender						
female	18851	44.08	4,817	45.06	0.068	0.020
male	23915	55.92	5,873	54.94	0.068	0.020
Age, year						
20-40	6,240	14.59	1,693	15.84	0	0.035
40-60	21561	50.42	5,442	50.91	0	0.010
>60	14965	34.99	3,555	33.26	0	0.037
Mean Age (SD)	54	12.91	53.33	13.04	0	0.052
Urbanization						
1 (high)	23609	55.21	6,018	56.30	0.240	0.022
2	16193	37.86	3,950	36.95	0.240	0.019
3	2,420	5.66	586	5.48	0.240	0.008
4 (low)	544	1.27	136	1.27	0.240	0
Enrollee category						
White collar	21956	51.34	5,573	52.13	0.336	0.016
Blue collar	12734	29.78	3,138	29.35	0.336	0.009
Others	8,076	18.88	1979	18.51	0.336	0.010
Comorbidities						
Hyperlipidemia	17170	40.15	4,310	40.32	0.749	0.004
Hypertension	20908	48.89	5,247	49.08	0.720	0.004
Coronary artery disease	11576	27.07	2,896	27.09	0.963	0.001
Chronic kidney disease	7,922	18.52	1972	18.45	0.855	0.002
Stroke	8,687	20.31	2,158	20.19	0.772	0.003
COPD	10129	23.68	2,535	23.71	0.950	0.001
Connective tissue disease	4,230	9.89	1,033	9.66	0.480	0.008
Heart failure	3,233	7.56	781	7.31	0.373	0.010
Malignancy	4,956	11.59	1,232	11.52	0.854	0.002
Liver cirrhosis	13898	32.5	3,476	32.52	0.971	0
Alcoholic liver disease	3,078	7.2	739	6.91	0.307	0.011
Obesity	1,215	2.84	284	2.66	0.302	0.011
aDCSI score						
0	5,201	12.16	1,328	12.42	0.386	0.008
1	4,917	11.5	1,183	11.07	0.386	0.014
≥2	32648	76.34	8,179	76.51	0.386	0.004
Medication						
Metformin	39194	91.65	9,412	88.04	<0.0001	0.120
TZD	13600	31.8	3,366	31.49	0.533	0.007
SGLT2 inhibitor	7,907	18.49	2054	19.21	0.085	0.019
acarbose	9,799	22.91	2,461	23.02	0.811	0.003
Sulfonylurea	31181	72.91	7,541	70.54	<0.0001	0.053
DPP-4 inhibitor	11938	27.91	2,783	26.03	<0.0001	0.042
Insulin	20690	48.38	5,159	48.26	0.825	0.002
Oral medication combination	6,404	14.97	1,481	13.85	0.003	0.032

Data shown as n(%) or mean ± SD.

SMD: standardized mean difference. A standardized mean difference of 0.1 or less indicates a negligible difference.

GLP1-RA, glucagon-like peptide-1, receptor agonist; COPD, chronic obstructive pulmonary disease; TZD, thiazolidinedione; SGLT2, Sodium-glucose co-transporter-2; DPP-4, inhibitor, dipeptidyl peptidase-4 inhibitor.

The urbanization level was categorized by the population density of the residential area into four levels, with level 1 as the most urbanized and level 4 as the least urbanized.

1:4 propensity score matching.

**FIGURE 2:**
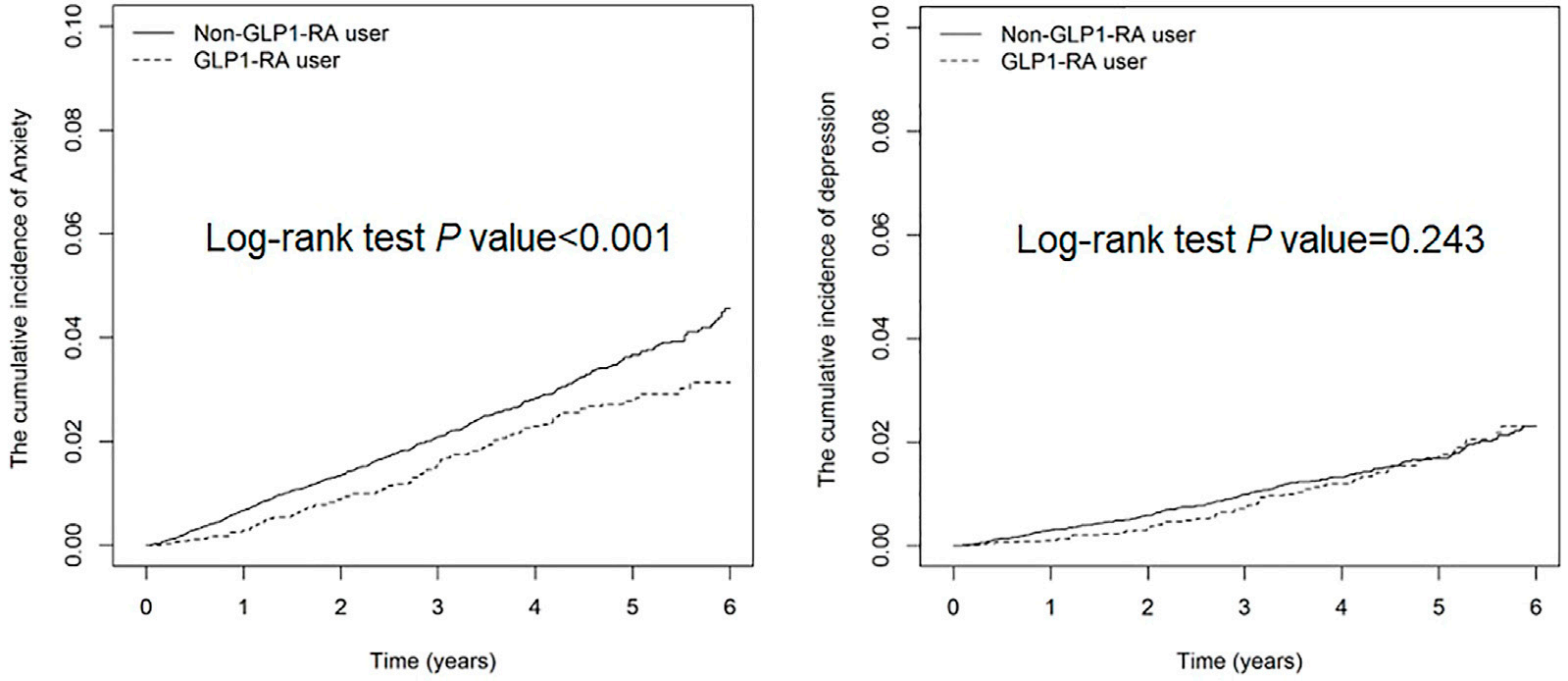
Cumulative incidence of depression and anxiety in diabetes patients with/without GLP1-RA.

The cumulative incidence of anxiety was 2.13% lower in GLP1-RA users than non-users (Log-rank test *p* < 0.001), whereas that of depression was not significantly different between the 2 groups (Figure 2). The overall incidence of depression and/or anxiety was lower in GLP1-RA users than non-users (6.80 *versus* 9.36 per 1,000 person-years), with an aHR of 0.8 (95% CI: 0.67–0.95) for users, after controlling for demographic factors, comorbidities and medications (Table 2). The difference of incidence rates between the two groups was greater for anxiety than depression. The aHRs of developing anxiety and depression for the GLP1-RA group, compared to non-users, were 0.78 (95% CI: 0.64–0.95) and 0.94 (95% CI: 0.72–1.23), respectively.

**TABLE 2:** Incidence of depression and anxiety in diabetes patients with/without GLP1-RA use.

Outcome variables	Non-GLP1-RA vs. GLP1-RA user	
	Non-GLP1-RA user	GLP1-RA user
Any depression or anxiety		
Follow-up years	1.97 ± 1.53	2.62 ± 1.51
Event	857	157
Person-year	91546	23090
Incidence Rate	9.36	6.80
Crude HR (95% CI)	ref.	0.72 (0.60, 0.85)***
Model 1	ref.	0.73 (0.61, 0.86)***
Model 2	ref.	0.73 (0.62, 0.87)***
Model 3	ref.	0.80 (0.67, 0.95)**
Anxiety		
Follow-up years	1.93 ± 1.52	2.39 ± 1.48
Event	656	116
Person-year	91838	23158
Incidence Rate	7.14	5.01
Crude HR (95% CI)	ref.	0.70 (0.57, 0.85)***
Model 1	ref.	0.70 (0.58, 0.86)***
Model 2	ref.	0.71 (0.58, 0.86)***
Model 3	ref.	0.78 (0.64, 0.95)*
Depression		
Follow-up years	2.08 ± 1.57	2.92 ± 1.60
Event	308	68
Person-year	92594.49	23304.4
Incidence Rate	3.32	2.91
Crude HR (95% CI)	ref.	0.85 (0.66, 1.11)
Model 1	ref.	0.85 (0.66, 1.11)
Model 2	ref.	0.87 (0.67, 1.14)
Model 3	ref.	0.94 (0.72, 1.23)

*p < 0.05, **p < 0.01, ***p < 0.001.

Incidence rate, per 1,000 person-years; HR, hazard ratio; CI, confidence interval.

Demographic factors include age, gender, urbanization level and enrollee category.

1:4 propensity score matching.

Model 1: Adjust for demographic factors.

Model 2: Adjust for model 1 + comorbidity.

Model 3: Adjust for model 2 + medication.

The beneficial effect on anxiety (Table 3) was specific to patients between 40 and 60 years old, with aHR of 0.73 (95% CI: 0.55–0.96). Compared with men, women in both groups exhibited greater incidences of depression and anxiety (Table 4). However, for women, risks of anxiety were significantly lower in GLP1-RA users than non-users. GLP1-RA users with either kind of comorbidity or that used other hypoglycemia agents showed no significant difference on the risk of depression (Table 4). On the other hand, GLP1-RA users without comorbidities except for hypertension showed significant reductions of anxiety (Table 3). GLP1-RA users with hypertension tended to have lower risk of anxiety, with aHR of 0.73 (95% CI: 0.54–0.97). GLP1-RA users that took metformin, sulfonylurea and oral medication combination had lower risk of anxiety, so as to those that did not use TZD, acarbose, SGLT2 inhibitors, DPP4 inhibitors, or insulin (Table 3).

**TABLE 3:** Factors associated with anxiety incidence in diabetes patients with/without GLP1-RA: subgroup analysis.

Variables	Non-GLP1-RA user			GLP1-RA user			cHR	(95% CI)	*p*-value	aHR	(95% CI)	*p*-value
	Event	PY	IR	Event	PY	IR						
Anxiety												
Gender												
Female	348	42,211	8.24	63	11,093	5.68	0.68	(0.52, 0.89)**	0.005	0.75	(0.57, 0.98)*	0.034
Male	308	49,627	6.21	53	12,065	4.39	0.71	(0.53, 0.94)*	0.019	0.80	(0.60, 1.08)	0.145
Age group												
20-40	82	12,988	6.31	24	4,519	5.31	0.87	(0.55, 1.37)	0.538	0.92	(0.57, 1.46)	0.713
40-60	328	47,119	6.96	59	12,642	4.67	0.66	(0.50, 0.87)**	0.003	0.73	(0.55, 0.96)*	0.027
>60	246	31,731	7.75	33	5,997	5.50	0.71	(0.50, 1.03)	0.071	0.75	(0.52, 1.09)	0.130
Comorbidities												
Hyperlipidemia												
No	381	54,417	7.00	67	14,265	4.70	0.66	(0.51, 0.85)**	0.002	0.72	(0.56, 0.94)*	0.014
Yes	275	37,422	7.35	49	8,893	5.51	0.76	(0.56, 1.03)	0.072	0.86	(0.63, 1.17)	0.328
Hypertension												
No	311	46,371	6.71	63	12,092	5.21	0.76	(0.58, 1.00)*	0.048	0.82	(0.62, 1.08)	0.157
Yes	345	45,467	7.59	53	11,066	4.79	0.63	(0.47, 0.85)**	0.002	0.73	(0.54, 0.97)*	0.033
Coronary artery disease												
No	469	66,416	7.06	77	17,076	4.51	0.63	(0.50, 0.80)***	<0.001	0.69	(0.54, 0.88)**	0.003
Yes	187	25,422	7.36	39	6,083	6.41	0.87	(0.62, 1.23)	0.441	1.01	(0.71, 1.44)	0.953
Chronic kidney disease												
No	519	75,604	6.86	94	19,379	4.85	0.7	(0.56, 0.87)**	0.001	0.77	(0.61, 0.96)*	0.018
Yes	137	16,234	8.44	22	3,779	5.82	0.69	(0.44, 1.09)	0.111	0.79	(0.50, 1.24)	0.303
Stroke												
No	510	73,191	6.97	87	18,674	4.66	0.66	(0.53, 0.83)***	<0.001	0.73	(0.58, 0.92)**	0.008
Yes	146	18,647	7.83	29	4,484	6.47	0.82	(0.55, 1.23)	0.343	0.93	(0.62, 1.39)	0.717
COPD												
No	488	69,597	7.01	81	17,840	4.54	0.64	(0.51, 0.81)***	<0.001	0.70	(0.55, 0.89)**	0.003
Yes	168	22,241	7.55	35	5,319	6.58	0.86	(0.60, 1.24)	0.429	0.99	(0.69, 1.44)	0.978
Connective tissue disease												
No	576	82,793	6.96	100	21,190	4.72	0.67	(0.54, 0.83)***	<0.001	0.74	(0.60, 0.92)**	0.006
Yes	80	9,045	8.84	16	1,969	8.13	0.91	(0.53, 1.57)	0.746	1.03	(0.59, 1.78)	0.921
Heart failure												
No	594	85,201	6.97	103	21,739	4.74	0.67	(0.55, 0.83)***	<0.001	0.75	(0.60, 0.92)**	0.007
Yes	62	6,637	9.34	13	1,420	9.16	0.98	(0.54, 1.78)	0.943	1.11	(0.60, 2.05)	0.744
Malignancy												
No	575	81,517	7.05	98	20,816	4.71	0.66	(0.53, 0.82)***	<0.001	0.73	(0.59, 0.90)**	0.004
Yes	81	10,321	7.85	18	2,343	7.68	0.97	(0.58, 1.62)	0.907	1.11	(0.66, 1.86)	0.703
Liver cirrhosis												
No	412	60,426	6.82	72	15,490	4.65	0.67	(0.52, 0.87)**	0.002	0.72	(0.56, 0.93)*	0.012
Yes	244	31,413	7.77	44	7,668	5.74	0.74	(0.54, 1.02)	0.066	0.85	(0.62, 1.18)	0.339
Alcoholic liver disease												
No	606	85,550	7.08	103	21,819	4.72	0.66	(0.54, 0.81)***	<0.001	0.73	(0.59, 0.90)**	0.003
Yes	50	6,288	7.95	13	1,340	9.70	1.21	(0.65, 2.22)	0.549	1.30	(0.69, 2.45)	0.411
Obesity												
No	632	89,423	7.07	111	22,657	4.90	0.69	(0.56, 0.84)***	<0.001	0.76	(0.62, 0.93)**	0.007
Yes	24	2,416	9.94	5	501	9.97	1	(0.38, 2.62)	0.997	1.28	(0.45, 3.66)	0.640
Medication												
Metformin												
No	65	5,843	11.12	16	1,567	10.21	0.9	(0.52, 1.55)	0.694	1.11	(0.62, 1.98)	0.721
Yes	591	85,995	6.87	100	21,591	4.63	0.67	(0.54, 0.82)***	<0.001	0.74	(0.60, 0.92)**	0.007
TZD												
No	463	56,264	8.23	82	14,733	5.57	0.68	(0.54, 0.86)**	0.001	0.77	(0.60, 0.97)*	0.027
Yes	193	35,575	5.43	34	8,425	4.04	0.72	(0.50, 1.04)	0.079	0.78	(0.54, 1.13)	0.189
Acarbose												
No	480	63,351	7.58	78	15,938	4.89	0.64	(0.50, 0.81)***	<0.001	0.72	(0.56, 0.91)**	0.007
Yes	176	28,487	6.18	38	7,221	5.26	0.85	(0.60, 1.20)	0.357	0.87	(0.61, 1.24)	0.443
SGLT2 inhibitor												
No	613	73,197	8.37	103	15,888	6.48	0.78	(0.63, 0.96)*	0.019	0.78	(0.63, 0.96)*	0.018
Yes	43	18,641	2.31	13	7,270	1.79	0.7	(0.37, 1.31)	0.263	0.66	(0.34, 1.26)	0.207
Sulfonylurea												
No	135	17,895	7.54	25	4,376	5.71	0.76	(0.49, 1.16)	0.207	0.84	(0.55, 1.31)	0.446
Yes	521	73,943	7.05	91	18,782	4.85	0.68	(0.54, 0.85)***	<0.001	0.75	(0.60, 0.95)*	0.014
DPP-4 inhibitor												
No	480	56,240	8.53	67	13,045	5.14	0.6	(0.47, 0.78)***	<0.001	0.65	(0.50, 0.84)**	0.001
Yes	176	35,598	4.94	49	10,114	4.85	0.91	(0.66, 1.25)	0.575	1.01	(0.73, 1.41)	0.939
Insulin												
No	283	37,285	7.59	45	8,647	5.20	0.69	(0.5, 0.94)*	0.019	0.68	(0.49, 0.93)*	0.015
Yes	373	54,554	6.84	71	14,511	4.89	0.7	(0.54, 0.90)**	0.005	0.82	(0.63, 1.06)	0.127
Oral medication combination												
No	580	74,490	7.79	100	18,767	5.33	0.68	(0.55, 0.85)***	<0.001	0.76	(0.62, 0.94)*	0.013
Yes	76	17,348	4.38	16	4,392	3.64	0.76	(0.44, 1.31)	0.321	0.88	(0.51, 1.53)	0.652

*p < 0.05, **p < 0.01, ***p < 0.001.

PY, person-years; IR, incidence rate, per 1,000 person-years; HR, hazard ratio; CI, confidence interval.

Demographic factors include age, gender, urbanization level and enrollee category.

1:4 propensity score matching.

Fully adjusted model: Adjusted for demographic factors, comorbidities and medication.

**TABLE 4:** Factors associated with depression incidence in diabetes patients with/without GLP1-RA: subgroup analysis.

Variables	Non-GLP1-RA user			GLP1-RA user			cHR	(95% CI)	*p*-value	aHR	(95% CI)	*p*-value
	Event	PY	IR	Event	PY	IR						
Depression												
Gender												
Female	169	42,634	3.96	39	11,180	3.49	0.85	(0.60, 1.21)	0.367	0.91	(0.64, 1.29)	0.594
Male	139	49,961	2.78	29	12,125	2.39	0.85	(0.57, 1.26)	0.414	0.95	(0.64, 1.43)	0.812
Age group												
20-40	48	13,071	3.67	20	4,538	4.41	1.13	(0.67, 1.91)	0.654	1.32	(0.77, 2.26)	0.316
40-60	141	47,504	2.97	30	12,723	2.36	0.77	(0.52, 1.15)	0.202	0.86	(0.58, 1.28)	0.461
>60	119	32,020	3.72	18	6,043	2.98	0.8	(0.49, 1.32)	0.392	0.8	(0.49, 1.32)	0.388
Comorbidities												
Hyperlipidemia												
No	162	54,908	2.95	40	14,355	2.79	0.9	(0.64, 1.28)	0.567	0.96	(0.67, 1.36)	0.798
Yes	146	37,687	3.87	28	8,949	3.13	0.81	(0.54, 1.21)	0.296	0.93	(0.62, 1.40)	0.726
Hypertension												
No	139	46,700	2.98	32	12,182	2.63	0.85	(0.58, 1.25)	0.416	0.87	(0.59, 1.28)	0.479
Yes	169	45,894	3.68	36	11,123	3.24	0.86	(0.60, 1.24)	0.428	1.01	(0.70, 1.45)	0.967
Coronary artery disease												
No	198	67,040	2.95	47	17,161	2.74	0.89	(0.64, 1.22)	0.457	0.93	(0.68, 1.29)	0.681
Yes	110	25,555	4.30	21	6,143	3.42	0.81	(0.50, 1.29)	0.366	0.94	(0.59, 1.51)	0.802
Chronic kidney disease												
No	228	76,269	2.99	54	19,496	2.77	0.89	(0.66, 1.20)	0.456	0.94	(0.70, 1.27)	0.689
Yes	80	16,325	4.90	14	3,808	3.68	0.76	(0.43, 1.34)	0.339	0.92	(0.52, 1.63)	0.773
Stroke												
No	225	73,841	3.05	51	18,776	2.72	0.86	(0.64, 1.17)	0.346	0.93	(0.69, 1.27)	0.668
Yes	83	18,754	4.43	17	4,528	3.75	0.84	(0.50, 1.42)	0.512	0.97	(0.57, 1.66)	0.925
COPD												
No	205	70,232	2.92	51	17,928	2.85	0.94	(0.69, 1.28)	0.689	1	(0.73, 1.36)	0.988
Yes	103	22,362	4.61	17	5,376	3.16	0.69	(0.41, 1.15)	0.151	0.83	(0.50, 1.4)	0.493
Connective tissue disease												
No	256	83,497	3.07	59	21,319	2.77	0.87	(0.66, 1.16)	0.349	0.95	(0.71, 1.27)	0.740
Yes	52	9,098	5.72	9	1986	4.53	0.8	(0.39, 1.63)	0.545	0.91	(0.44, 1.87)	0.792
Heart failure												
No	269	85,902	3.13	62	21,875	2.83	0.87	(0.66, 1.15)	0.343	0.95	(0.72, 1.25)	0.697
Yes	39	6,692	5.83	6	1,429	4.20	0.75	(0.32, 1.78)	0.516	0.9	(0.37, 2.17)	0.817
Malignancy												
No	251	82,226	3.05	57	20,935	2.72	0.87	(0.65, 1.15)	0.325	0.93	(0.70, 1.25)	0.636
Yes	57	10,369	5.50	11	2,369	4.64	0.84	(0.44, 1.60)	0.590	1	(0.52, 1.92)	0.996
Liver cirrhosis												
No	171	60,946	2.81	44	15,577	2.83	0.97	(0.70, 1.35)	0.854	1	(0.72, 1.40)	0.995
Yes	137	31,649	4.33	24	7,728	3.11	0.71	(0.46, 1.10)	0.126	0.85	(0.54, 1.32)	0.459
Alcoholic liver disease												
No	271	86,283	3.14	65	21,946	2.96	0.91	(0.69, 1.20)	0.503	0.98	(0.74, 1.29)	0.887
Yes	37	6,311	5.86	3	1,358	2.21	0.4	(0.12, 1.28)	0.122	0.47	(0.14, 1.57)	0.221
Obesity (numbers of samples in these cell were less than 3 and therefore cannot be compared)												
No	297	90,162	3.29	68	22,796	2.98	0.88	(0.68, 1.15)	0.343	0.96	(0.74, 1.25)	0.768
Yes	11	2,433	4.52	0	NA	NA	NA	NA	NA	NA	NA	NA
Medication												
Metformin												
No	37	5,911	6.26	5	1,600	3.13	0.51	(0.20, 1.29)	0.155	0.53	(0.19, 1.42)	0.206
Yes	271	86,684	3.13	63	21,704	2.90	0.9	(0.68, 1.18)	0.441	0.98	(0.74, 1.30)	0.908
TZD												
No	221	56,829	3.89	46	14,832	3.10	0.78	(0.57, 1.07)	0.124	0.89	(0.65, 1.23)	0.479
Yes	87	35,765	2.43	22	8,472	2.60	1.03	(0.65, 1.65)	0.891	1.06	(0.65, 1.71)	0.821
Acarbose												
No	225	63906	3.52	45	16,044	2.81	0.77	(0.56, 1.06)	0.112	0.88	(0.63, 1.21)	0.427
Yes	83	28689	2.89	23	7,260	3.17	1.08	(0.68, 1.71)	0.749	1.08	(0.67, 1.74)	0.737
SGLT2 inhibitor												
No	285	73,937	3.85	54	16,036	3.37	0.88	(0.66, 1.18)	0.387	0.87	(0.65, 1.17)	0.362
Yes	23	18,657	1.23	14	7,268	1.93	1.21	(0.62, 2.37)	0.579	1.1	(0.55, 2.22)	0.780
Sulfonylurea												
No	69	18,015	3.83	17	4,402	3.86	1.02	(0.60, 1.73)	0.950	1.24	(0.72, 2.14)	0.440
Yes	239	74,579	3.20	51	18,903	2.70	0.81	(0.60, 1.10)	0.177	0.86	(0.64, 1.18)	0.353
DPP-4 inhibitor												
No	211	56,832	3.71	33	13,138	2.51	0.68	(0.47, 0.98)*	0.040	0.75	(0.52, 1.09)	0.132
Yes	97	35,762	2.71	35	10,167	3.44	1.17	(0.79, 1.73)	0.424	1.23	(0.82, 1.84)	0.310
Insulin												
No	112	37,656	2.97	23	8,708	2.64	0.91	(0.58, 1.43)	0.682	0.93	(0.59, 1.47)	0.765
Yes	196	54,938	3.57	45	14,597	3.08	0.82	(0.59, 1.14)	0.237	0.93	(0.67, 1.30)	0.685
Oral medication combination												
No	267	75,194	3.55	61	18,888	3.23	0.89	(0.68, 1.18)	0.428	0.98	(0.74, 1.30)	0.908
Yes	41	17,400	2.36	7	4,416	1.59	0.61	(0.27, 1.37)	0.233	0.66	(0.29, 1.50)	0.319

*p < 0.05, **p < 0.01, ***p < 0.001.

PY, person-years; IR, incidence rate, per 1,000 person-years; HR, hazard ratio; CI, confidence interval.

Demographic factors include age, gender, urbanization level and enrollee category.

1:4 propensity score matching.

Fully adjusted model: Adjusted for demographic factors, comorbidities and medication.

The incidence of depression or anxiety decreased with increasing duration of treatment after the initiation of GLP1-RA medication (Table 5). The reduction trends were significant after controlling for all covariates. After taking the medicine for 180 days or longer, rates of incidence of depression or anxiety reduced to 2.19 and 2.93 per 1,000 person-years, respectively. The risk of having any depression or anxiety fell to an aHR of 0.5 (95% CI: 0.36–0.69) in GLP1-RA users, compared to non-users. We also evaluated the effect of different GLP1-RA on anxiety or depression in Tables 6 and 7. Our results revealed that dulaglutide could significantly reduce risks of anxiety and depression, while liraglutide and exenatide showed no significant reductions on risks of either anxiety or depression.

**TABLE 5:** Risk of depression and anxiety associated with duration of GLP1-RA use.

Variables	Any depression or anxiety (n = 1,014)			cHR	(95% CI)	aHR	(95% CI)
	Event	PY	IR				
non-use GLP1-RA	857	91,546	9.36	1.00	(Reference)	1.00	(Reference)
GLP1-RA drug days							
30–90 days	72	7,542	9.55	1.01	(0.79, 1.29)	1.22	(0.96, 1.56)
91–180 days	47	7,380	6.37	0.68	(0.5, 0.91)**	0.76	(0.57, 1.02)
>180	38	8,169	4.65	0.49	(0.35, 0.67)***	0.5	(0.36, 0.69)***

Variables	Anxiety (*n* = 772)			cHR	(95% CI)	aHR	(95% CI)
	Event	PY	IR				
non-use GLP1-RA	656	91,838	7.14	1.00	(Reference)	1.00	(Reference)
GLP1-RA drug days							
30–90 days	56	7,570	7.40	1.03	(0.79, 1.36)	1.3	(0.98, 1.71)
91–180 days	36	7,403	4.86	0.68	(0.48, 0.95)*	0.77	(0.55, 1.07)
>180 days	24	8,185	2.93	0.4	(0.27, 0.61)***	0.41	(0.27, 0.61)***

Variables	Depression (*n* = 376)			cHR	(95% CI)	aHR	(95% CI)
	Event	PY	IR				
non-use GLP1-RA	308	92,594	3.33	1.00	(Reference)	1.00	(Reference)
GLP1-RA drug days							
30–90 days	32	7,629	4.19	1.24	(0.86, 1.78)	1.4	(0.97, 2.03)
91–180 days	18	7,460	2.41	0.71	(0.44, 1.15)	0.79	(0.49, 1.27)
>180 days	18	8,215	2.19	0.63	(0.39, 1.02)	0.68	(0.42, 1.10)

PY, person-years; IR, incidence rate, per 1,000 person-years; HR, hazard ratio; CI, confidence interval.

Demographic factors include age, gender, urbanization level and enrollee category1:4 propensity score matching.

**TABLE 6:** Cox proportional hazard model estimated GLP1-RA hazard ratio of anxiety among different GLP1-RA types.

Variables	Anxiety			cHR	(95% CI)	aHR^#^	(95% CI)
	n	PY	IR				
Non-use GLP1-RA	688	102,030	6.74	1.00	(Reference)	1.00	(Reference)
Liraglutide	84	129,66	6.48	0.91	(0.72, 1.14)	1.07	(0.85, 1.35)
Non-use GLP1-RA	741	110,339	6.72	1.00	(Reference)	1.00	(Reference)
Exenatide	31	4,658	6.66	0.92	(0.64, 1.33)	1.3	(0.90, 1.89)
Non-use GLP1-RA	750	105,355	7.12	1.00	(Reference)	1.00	(Reference)
Dulaglutide	22	9,642	2.28	0.34	(0.22, 0.52)***	0.32	(0.21, 0.49)***

PY: person-years; IR: incidence rate per 1,000 person-years; cHR: crude hazard ratio; aHR: adjusted hazard ratio.

1:4 propensity score matching.

# : Adjusted by sex, age, urbanization level, enrollee category, comorbidities and medication.

*p < 0.05, **p < 0.01, ***p < 0.001.

**TABLE 7:** Cox proportional hazard model estimated GLP1-RA hazard ratio of depression among different GLP1-RA types.

Variables	Depression			cHR	(95% CI)	aHR^#^	(95% CI)
	n	PY	IR				
Non-use GLP1-RA	329	102,822	3.20	1.00	(Reference)	1.00	(Reference)
Liraglutide	47	13,077	3.59	1	(0.73, 1.36)	1.13	(0.82, 1.54)
Non-use GLP1-RA	356	111,188	3.20	1.00	(Reference)	1.00	(Reference)
Exenatide	20	4,711	4.25	1.13	(0.71, 1.78)	1.43	(0.90, 2.28)
Non-use GLP1-RA	363	106,247	3.42	1.00	(Reference)	1.00	(Reference)
Dulaglutide	13	9,652	1.35	0.44	(0.25, 0.78)**	0.45	(0.26, 0.79)**

PY: person-years; IR: incidence rate per 1,000 person-years; cHR: crude hazard ratio; aHR: adjusted hazard ratio.

1:4 propensity score matching.

# : adjusted by sex, age, urbanization level, enrollee category, comorbidities and medication; *p < 0.05, **p < 0.01, ***p < 0.001.

## Discussion

To our knowledge, this study represents the largest population-based analyses investigating whether GLP1-RA medication is associated with reduced risks of depression or anxiety in DM patients. GLP1-RA users exhibited a significant risk reduction for anxiety, and a moderate reduction for depression, compared with non-users. This treatment effectiveness on anxiety was observed in female users but not in male users. The effectiveness increased with the duration of medication and the significant risk reduction was observed after 6-months or longer therapy. Further age-specific stratified analyses revealed significant reduction of anxiety in patients between 40 and 60 years. GLP1-RA users taking metformin or sulfonylurea at the same time had decreased risk of anxiety, which was also noted in patients with hypertension. As for the specific effectiveness of each GLP1-RA, dulaglutide use could significantly decrease risks of both anxiety and depression, while liraglutide and exenatide showed no significant effect on reductions of either anxiety or depression.

Our findings on reduced risks of anxiety or depression associated with GLP1-RA use were consistent with some previous findings (Bode et al., 2010; Grant et al., 2011; Idris et al., 2013; de Wit et al., 2014), but contrast with others that showed no significant association between GLP1-RA medication and depression (de Wit et al., 2016; O'Neil et al., 2017; Kahal et al., 2019). However, studies with non-significant findings were either based on small sample sizes or were not focused on DM patients. Although Eren-Yazicioglu et al. (2021) reported that exenatide was associated with higher depressive scores indirectly through its effect on perceived stress, their study participants already had lifetime or current psychiatric diagnosis at baseline, which was different from our study (we excluded those with prior anxiety or depression diagnosis at baseline).

Neuroinflammation might be a critical factor for the onset, deterioration, relapse, and maintenance of depression or anxiety (Kopschina Feltes et al., 2017; Paudel et al., 2018; Woelfer et al., 2019). GLP-1 has been reported to promote productions of anti-inflammatory cytokines in various organs, including the adipose tissue, the pancreas, and the brain (Dobrian et al., 2011; Lee et al., 2012; Darsalia et al., 2014; Augestad et al., 2020; Reed et al., 2020). Kim et al. (2020) summarized that GLP1-RA may ameliorate depression by reducing neuroinflammation, balancing neurotransmitter homeostasis, promoting neuronal differentiation and neural stem cell proliferation and improving synaptic function. Albeit we failed to find significant reductions in the overall incidence of depression in GLP1-RA users, our subgroup analysis showed that patients using dulaglutide were at a lower risk of depression than non-users. GLP1-RA treatment-associated decrease in the risk of depression or anxiety in DM patients may be driven by combinations of anti-inflammation, better glycemic control, greater weight loss, and reduced concern about weight gain (Bode et al., 2010).

Women are generally more likely than men to develop an anxiety disorder (Leach et al., 2008; McLean and Anderson, 2009). The present study demonstrated that women were also at a higher incidence of anxiety risk than men even after using GLP1-RA. However, female users had a greater benefit from GLP1-RA therapy, than male users. The treatment effectiveness of GLP1-RA might be due to interactions between GLP-1R, the central and peripheral nervous system, estrogen, and the anorexic actions. GLP-1 is secreted from gut enteroendocrine cells and brain preproglucagon (PPG) neurons, which are known as the peripheral and central GLP-1 systems (Brierley et al., 2021). GLP-1 secreted by the intestine releases into the hepatoportal vein. This activates the vagus nerve to generate a neural signal towards the brain stem, such as the nucleus of the solitary tract nucleus (NTS) and the area postrema (AP), which send axons to the hypothalamus to release GLP-1 and activate the receptors. A new signal is then sent towards peripheral tissues through the autonomic nervous system (ANS) to regulate numerous functions (Cabou and Burcelin, 2011). However, research indicated that the intake-inhibitory effects of the GLP-1 RA, exendin-4 and liraglutide, were mediated by activation of GLP-1R expressed on sub-diaphragmatic vagal afferents as well as in the brain (Kanoski et al., 2011). That is, central and peripheral GLP-1 systems suppress eating *via* independent gut-brain circuits (Brierley et al., 2021). Central injections of GLP1-RA strongly decrease food intake, due to GLP-1R expression targeting the hypothalamic and brainstem inducing anorexic action. (Larsen et al., 1997; McMahon and Wellman, 1998; Hayes et al., 2008; Hayes et al., 2010; Kanoski et al., 2011). The same CNS regions are implicated for the anorexic action of estrogens (Palmer and Gray, 1986; Osterlund et al., 1998; Merchenthaler et al., 2004; Musatov et al., 2007), and may provide neuroanatomical grounds for the interaction between GLP-1 receptor and gender (Richard et al., 2016) in our results. Such a mechanism was also supported by Richard et al. (2016), who reported that women are more sensitive than men to the food reward impact of central GLP-1 receptor activation. In addition, individuals with obesity and overweight are more likely to have anxiety than non-obese persons (Amiri and Behnezhad, 2019). Since GLP1-RA is well known for its effect on weight loss, this might lead to the elimination of anxiety in women. Furthermore, younger DM patients with fewer comorbidities may be more concerned about body weight. They were probably more willing to adhere to the prescription of GLP1-RA and thus the risk of anxiety might be decreased.

Our study demonstrated that the combination of GLP1-RA with metformin exerted an anxiolytic effect, which was consistent with previous preclinical studies (Fan et al., 2019; Ji et al., 2019; Zemdegs et al., 2019; Turan et al., 2021). We also found that GLP1-RA users taking sulfonylurea had lower risk of anxiety, which may be due to the lower dose of sulfonylurea needed after using GLP1-RA, which could decrease the hypoglycemic risk of sulfonylurea. In our study, GLP1-RA users not using TZD also had a reduced risk of anxiety. This might be owing to the lowered side effect of weight gain from not using TZD. The finding that GLP1-RA users not using DPP-4 inhibitors had better reduction of anxiety was somewhat surprising. Numerous studies have reported that patients with mood and anxiety-related disorders are characterized by increased circulating inflammatory cytokines, including interleukin (IL)-1, IL-6, tumor necrosis factor (TNF), their soluble receptors, and acute phase reactants such as C-reactive protein (CRP) (Maes et al., 1992; Maes, 1999; Sluzewska, 1999; Michopoulos et al., 2017). DPP-4 is a novel adipokine secreted from adipose tissue (Lamers et al., 2011) with a pro-inflammatory role (Cordero et al., 1997). Obese patients may have higher DPP-4 levels from adipose tissue, and DPP-4 might induce depression by activating the immune system and promoting proinflammatory cytokine secretion. Previous studies revealed that although very low levels of the DPP-4 inhibitors were found in the brain (Fuchs et al., 2009; Fura et al., 2009), its neuroprotective effects might be more attributed to peripheral functions rather than directly in the central nervous system (Darsalia et al., 2013; Lin and Huang, 2016). On the other hand, GLP-1 RA crossed the BBB successfully (Hunter and Hölscher, 2012; Athauda and Foltynie, 2016). In Taiwan, combination usage of GLP1-RA and DPP-4 inhibitor is not reimbursed by health insurance. That is, either kind of medication has to be paid by the patient himself/herself. This may be another reason why GLP1-RA users not using DPP-4 inhibitors had better reduction of anxiety, since when the patient needs to pay for the other hypoglycemic agent, it may indicate that his or her glucose control is poorer, and may have more anxiety regarding health conditions compared to those who did not combine DPP-4 inhibitor with GLP1-RA.

Our study found different effects from each subtype of GLP1-RA on anxiety or depression, and dulaglutide was the only subtype associated with significant reduction in such risks. The distinct result may be due to differences in molecular structure that affects the potency, duration, and frequency of use (Monami et al., 2009; Buse et al., 2011). Dulaglutide may have better reductions of anxiety or depression due to its weekly injection formulation, when compared with liraglutide (daily injection). Exenatide and liraglutide also showed neuroprotective effect in few clinical studies among patients with Parkinson’s disease, Alzheimer’s disease, ischemia, traumatic brain injury, neuropathies, neurogenesis, or epilepsy, but not in patients with anxiety disorder as in our study. Erbil et al. (2019) suggested that not all GLP1-RAs have the same neuroprotective effect and the effect of GLP1-RAs on reversing neurodegenerative or neuro-destructive processes might be time and dose-dependent. Behavioral assessments in these preclinical studies are limited to the lesion model and may not cover all aspects of cortical function and all cortical layers. Hence, more studies investigating cortical effects of different subtypes of GLP-1 on depression or anxiety at the whole brain level, with longer assessment periods, and complete neuropsychological evaluations or behavioral observations are still needed both for preclinical and clinical studies.

The strength of this study lies in the very large number of patients used, the largest to-date, that focused on anxiety or depression associated with using GLP1-RA for the treatment for DM. We were able to conduct a longer duration follow-up than previous studies. The stratified data analysis was able to evaluate factors associated with treatment effectiveness. The identification of anxiety and depression were based on at least two clinical diagnoses rather than by screening instruments. However, several limitations should be acknowledged for this study. First, the study was observational, rather than a randomized control design, for the population of Taiwan. The generalizability of findings to other population may be restricted. Second, our efforts to match and adjust for possible confounding factors might be biased by unmeasured or unknown confounders not available in the NHIRD. Information on some potential confounders, including body weight, smoking, diet, exercise, stress, or family history, was not available in the NHIRD, the bias associated with unmeasured factors was unclear, despite our efforts to match study groups and controlling for available variables. Third, information on changes in body weight was unavailable in the NHIRD, we were unable to evaluate risks of anxiety or depression related to body weight changes.

## Conclusion

This population-based study demonstrated that recipients of GLP1-RA treatment for DM were at a significantly decreased risk of anxiety. However, the effectiveness was significant only for female users and patients using the medicine for longer than 180 days. The treatment effectiveness was moderate in reducing depression risk, especially for patients that used dulaglutide. These findings strengthen previous research advocating the antidepressant or anxiolytic effects of GLP1-RA.

## Data Availability

The original contributions presented in the study are included in the article/Supplementary Material, further inquiries can be directed to the corresponding author.
