# Corrigendum: Decreased Risk of Anxiety in Diabetic Patients Receiving Glucagon-Like Peptide-1 Receptor Agonist: A Nationwide, Population-Based Cohort Study

**DOI:** 10.3389/fphar.2022.886343

**Published:** 2022-03-22

**Authors:** Wen-Hsuan Tsai, Fung-Chang Sung, Lu-Ting Chiu, Ying-Hsiu Shih, Ming-Chieh Tsai, Shu-I Wu

**Affiliations:** ^1^ Division of Endocrinology and Metabolism, Department of Internal Medicine, Mackay Memorial Hospital, Taipei, Taiwan; ^2^ Management Office for Health Data (DryLab), Clinical Trial Research Center (CTC), China Medical University Hospital, Taipei, Taiwan; ^3^ Department of Health Services Administration, China Medical University, College of Public Health, Taichung, Taiwan; ^4^ Department of Food Nutrition and Health Biotechnology, Asia University, Taichung, Taiwan; ^5^ Department of Medicine, Mackay Medical College, New Taipei City, Taiwan; ^6^ Department of Psychiatry, Mackay Memorial Hospital, Taipei, Taiwan

**Keywords:** neuroendocrine, diabetes, glucagon-like peptide-1 receptor agonist, depression, anxiety

In the original article, there was an error in the **Results** section. The following paragraph was written incorrectly:

Both groups were similar in age and gender distributions, with a mean age of 53.33 years (SD 13.04) and 45.06% women (**Table 1**). Most baseline prevalence rates of comorbidities were significantly higher in GLP1-RA users than non-users, except for malignancy and alcoholic liver disease. The uses of other hypoglycemic agents were more prevalent in GLP1-RA users than non-users. The GLP1-RA group also had higher aDCSI scores, higher percentages of white-collar jobs and lived more in more urbanized areas.

This paragraph has been corrected to read as follows:

“Both groups had similar distributions of age, gender, occupation, urbanization and comorbidities, with a mean age of 53.33 years (SD 13.04) and 45.06% women (**Table 1**). Although, the percentage of using metformin was higher in non-GLP1-RA users.”

The authors apologize for this error and state that this does not change the scientific conclusions of the article in any way. The original article has been updated.

